# Magnesium phosphates experienced high-temperature transition found on the CI-like carbonaceous chondrite Yamato 980115 by Raman microspectroscopy

**DOI:** 10.1007/s44211-025-00720-0

**Published:** 2025-01-31

**Authors:** Hiroharu Yui, Hayato Tsychiya, Aruto Kashima, Shu-hei Urashima, Kenichi Oguchi, Naoya Imae, Akira Yamaguchi

**Affiliations:** 1 Department of Chemistry, Faculty of Science, Tokyo University of Science (TUS), 1-3 Kagurazaka, Shinjuku, Tokyo 162-8601 Japan; 2 Water Frontier Research Center, Research Institute for Science & Technology, Tokyo University of Science (WaTUS), 1-3 Kagurazaka, Shinjuku, Tokyo 162-8601 Japan; 3 National Institute of Polar Research (NIPR), 10-3 Midori-Cho, Tachikawa, Tokyo 190-8518 Japan; 4 The Graduate University for Advanced Studies (SOKENDAI), 10-3 Midori-Cho, Tachikawa, Tokyo 190-8518 Japan; 5 Present Address: Nuclear Science and Engineering Center, Japan Atomic Energy Agency (JAEA), 2-4 Shirakata, Tokai, Naka, Ibaraki 319-1195 Japan

**Keywords:** Antarctic meteorites, Carbonaceous chondrite, Magnesium phosphate, Thermal alteration, Raman spectroscopy

## Abstract

Magnesium phosphate grains, minor accessory minerals found on the primitive meteorite Yamato 980115 (Y 980115), were investigated by Raman microspectroscopy. All magnesium phosphate grains found in the present study can be assigned to farringtonite, dehydrated magnesium phosphate Mg_3_(PO_4_)_2_-I. Since the Mg_3_(PO_4_)_2_-I is generally formed via the irreversible thermal transition from the polymorphs of Mg_3_(PO_4_)_2_–II and –III at above 750–800 degree Celsius, we can infer that the parent body of the Y 980115 meteorites experienced thermal alteration with such a high temperature. This result is in good accordance with the previous studies and the proposals on the alteration history of Y 980115 by the electron-beam microscope and X-ray diffraction analyses. Furthermore, the hydrated form of the magnesium phosphates of Mg_3_(PO_4_)_2_·4H_2_O, Mg_3_(PO_4_)_2_·8H_2_O, and Mg_3_(PO_4_)_2_·22H_2_O was not found in the present research, also suggesting that the extensive vaporization of the hydrated water molecules with magnesium phosphate occurred by such high-temperature thermal alteration. Since Y 980115 has been historically categorized to heavily aqueously altered CI (Ivuna-type) carbonaceous chondrites but has distinct characteristic to CI meteorites, the present result would provide further evidence to the complexed alteration history of the parent body of Y 980115 meteorite.

**Graphical abstract:**

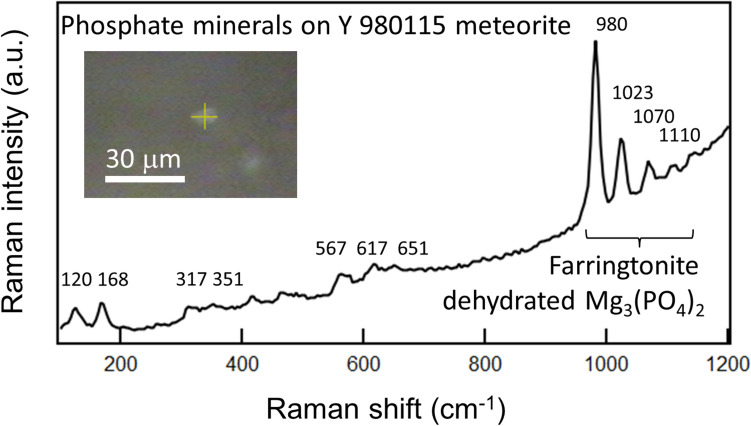

## Introduction

Analyses on chemical compounds and mineral crystals found on meteorites provide many crucial evidences and important clues for elucidating the chemical and material evolutions occurred at the solar system. Among them, carbonaceous chondrites carry various carbon materials and important geochemical information on the evolution of carbon materials at the early stage of the solar system formation [1]. Especially, Ivuna-type carbonaceous chondrites (CI) have been known that their parent bodies experienced heavy aqueous alteration. Thus, CI type carbonaceous chondrites have been investigated extensively to elucidate the water–carbon interactions that have been believed to provide organic molecules and carbon materials that relate to the fundamental biomolecules such as amino acids and nucleic acids. The directly collected samples by the Hayabusa2 mission (JAXA) from Asteroid 162173 Ryugu were also categorized basically to CI type and has been intensively studied [see, for example, 2–5]. We have also studied secondary minerals such as carbonates and pyrrhotites found on Ryugu particles by Raman microspectroscopy to investigate the aqueous alteration environments occurred on the parent body of asteroid Ryugu [6, 7].

In general, CI type carbonaceous chondrites show non-heated features above 150 degree Celsius (423 K), prevented from thermal alteration on chemical compounds and minerals found in them [1]. To the contrast, Yamato 980115 (Y 980115) meteorite, a carbonaceous chondrite collected from Yamato Mountains at East Antarctica in 1998, has been tentatively categorized to CI-like, but has distinct characteristics in geochemical point of view. Thus, Y 980115 has been also classified as a new category CY (“Yamato”) type [8] and has been investigated intensively in terms of the ages, chemical compositions, and thermal alteration of its parent body [9–12]. In terms of chemistry and mineralogy, the incomplete dehydration nature of Y 980115 was intensively investigated by using an optical microscopic observation, a field-emission scanning electron microscope (FE-SEM) equipped with an energy dispersive X-ray spectroscopy (EDS), an electron probe micro-analyzer (EPMA), and synchrotron X-ray diffraction (S-XRD) [13]. It was also suggested that the parent body of Y 980115 experienced heating above 500 degree Celsius using thermogravimetric analysis (TGA) and infrared spectroscopy (FT-IR) [14] and X-ray diffraction (XRD) [15].

Here, we report the Raman microspectroscopic study on Y 9800115, especially focusing on phosphate minerals. Various types of phosphate minerals have been expected to be sensitive indicators for aqueous conditions where they formed [16 and references therein]. Raman scattering spectra provide quite fruitful information on the slight difference in similar crystal structures and even in the differences in the number of hydrated water molecules in the crystals. We expected that the Raman spectroscopic study on the phosphate minerals found on Y 980115 would provide further insight and detailed thermodynamic and aqueous conditions of its parent body that were investigated by other various analytical tools mentioned above [8–15].

## Experimental

Four particles of Y 980115 were provided by National Institute of Polar Research (NIPR) and analyzed as received using Raman microscope system (Raman-11i, Nanophoton). The wavelength of the excitation laser was 532 nm. The excitation power was 1.0 mW at the sample faces of the meteorites. The incident laser was focused onto the samples with an objective lens (Plan fluor, NA 0.75, ×40, Nikon). Slit-width of the spectrometer in the Raman microscope system was 50 μm. The wavenumber of the spectra was calibrated with an intense Raman band (520.6 cm^−1^) from a silicon wafer surface. When we found a white, crystal-shaped grain with flat surface and a size of several tens of micrometers under optical microscopic observation, we acquired its Raman spectra as a single shot with the accumulation duration of 30 s. Among c.a. 600 times measurements on such grains, almost all of them were assigned to carbonates that showed the most intensive Raman peak at 1080–1100 cm^−1^ derived from the CO_3_^2−^ symmetric vibration. However, three grains clearly showed the sharp and intense peak at c.a. 960–980 cm^−1^ derived from the PO_4_^3−^ symmetric vibration that is characteristic to phosphate minerals. The acquired Raman spectra were shown as they were in the present manuscript; namely, no spectral processing was carried out such as a background reduction and an averaging of several data on the same point. This is because sometimes seeming background signal has important information on the chemistry of matrix silicate and each natural grain has its original history of its formation and alteration environments.

## Results and discussion

**Fig. 1:**
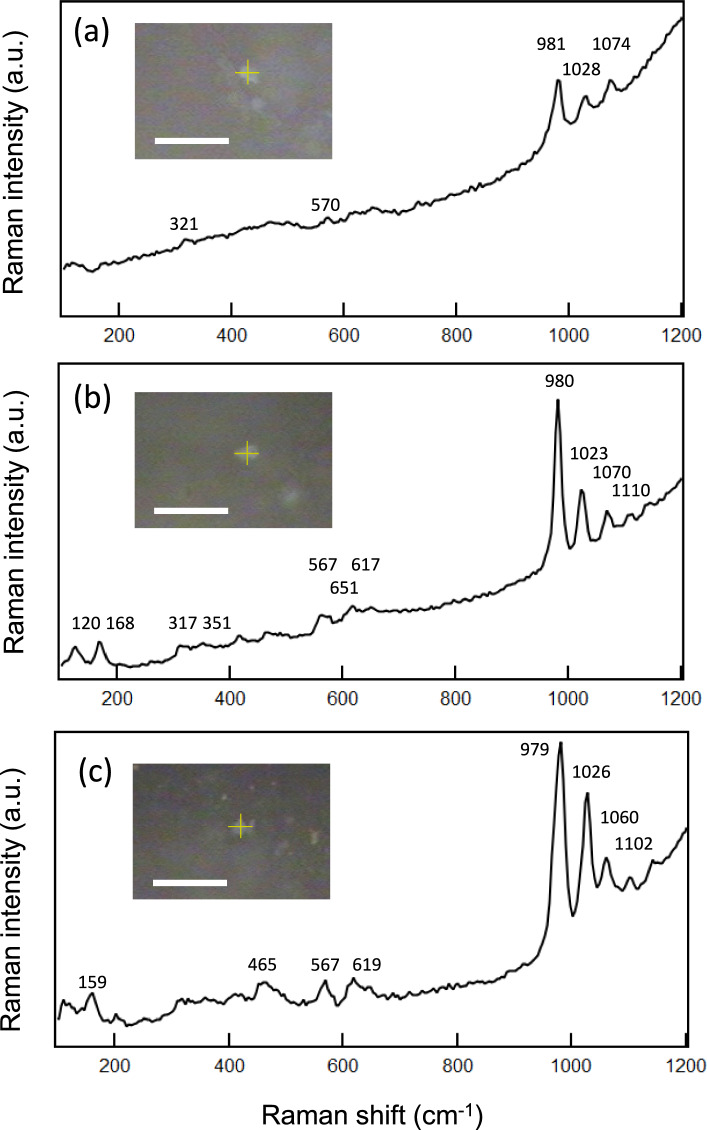
Raman spectra of the three grains of phosphate minerals found on Y 980115 meteorite. Insets are corresponding figures of the measured grains (scale bars: 30 μm)

In general, phosphates are minor accessory minerals and are rarely found on primitive meteorites such as CI chondrites. In the present research, we found three phosphate grains on Y 980115 particles. Figure 1 shows the optical pictures and their corresponding Raman spectra of the three phosphate grains. All of them show the most intense and characteristic Raman peak of PO_4_^3−^-stretching vibration at 960–980 cm^−1^, that is an important indicator for finding and assigning them to be phosphate minerals. In addition, above 1000 cm^−1^, we can see two or three intense Raman peaks that are assignable to other modes of PO_4_^3−^-stretching vibration.

**Fig. 2:**
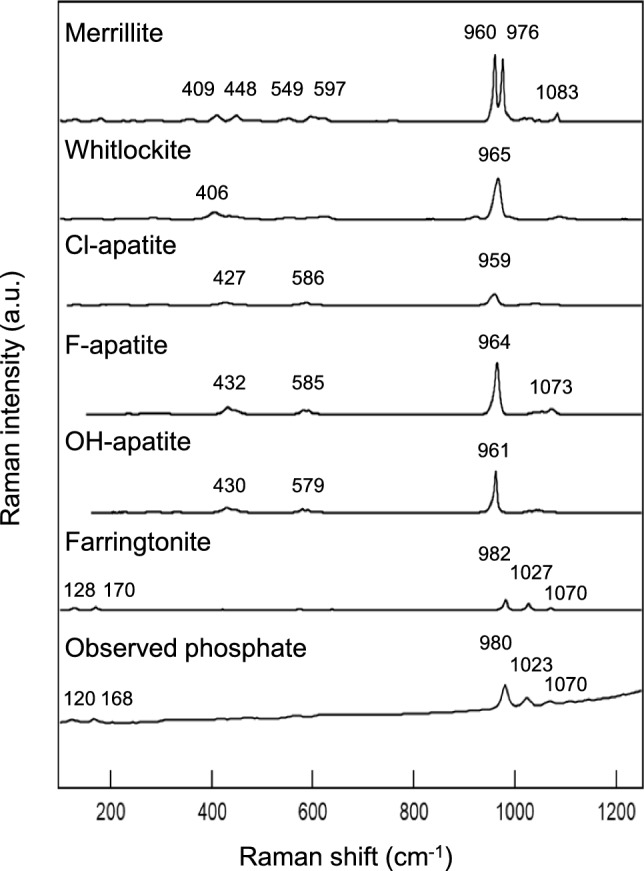
Comparisons of the Raman spectrum of the phosphate grain found on Y 980115 (Fig. 1**b**) and representative phosphate minerals (reconstructed from RRUFF data). The RRUFF data ID numbers are as follows. Merrillite (R150063)**,** whitlockite (R080052), Cl-apatite (R060192), F-apatite (R050529), OH-apatite (R100225), and farringtonite (R130127)

There are many kinds of phosphate minerals, and then, we assigned the kind of phosphate observed here by comparing Raman spectra of representative phosphate minerals found on both Earth and space environments including the moon and Mars [16]. Figure 2 compares the various kinds of representative phosphate minerals found on both Earth and space environments. Interestingly, the most intense Raman peaks of the observed phosphate grains showed remarkable higher shift (c.a. 980 cm^−1^) compared to those generally observed ones of representative phosphate (c.a. 960 cm^−1^). Among various kinds of phosphate minerals, the Raman shifts and spectral characteristics of phosphate grains found on Y 980115 showed good coincidence with of those of farringtonite. Farringtonite is the dehydrated form of magnesium phosphate Mg_3_(PO_4_)_2,_ which was found and characterized in pallasite meteorites [17, 18]. Pallasite meteorites are considered to be pieces derived from the core-mantle boundary of a small-sized planet, where a thermodynamic environment with considerable high-temperature (ca. > 800 degree Celsius) and also high pressure can be expected [19]. It is also known that farringtonite has three polymorphologies (-I, -II, -III) in their crystal structures and they were investigated by both synchrotron X-ray diffraction and Raman spectroscopy [20, 21].

To obtain further information on the thermodynamic conditions of the observed farringtonite grains on Y 980115, then we investigated its polymorphs. The Raman shifts of the most intensive Raman peaks of the polymorphs of Mg_3_(PO_4_)_2_-II and Mg_3_(PO_4_)_2_-III were reported to appear at around 960 cm^−1^ and their spectral patterns were different with that of Mg_3_(PO_4_)_2_-I [20, 21]. In addition, it is known that the temperature-induced phase transitions of Mg_3_(PO_4_)_2_-II and Mg_3_(PO_4_)_2_-III to Mg_3_(PO_4_)_2_-I occurring at 750–800 degree Celsius (1023–1073 K) are irreversible [20]. Since the characteristics of the Raman spectra of farringtonite grains found on Y 980115 show good coincidence with those of Mg_3_(PO_4_)_2_-I, we can infer that they experienced such high-temperature environments on their parent body of Y 980115 after their formation and precipitation in its aqueous environments. This observation is in good accordance with the suggestion and the consideration from the chemical and mineralogical investigations by TGA, electron-beam microscopes, and X-ray diffraction that have been previously reported [13–15]. Thus, the present Raman microscopic observation on phosphate grains provides further support to the proposals for the thermodynamic events occurred on the parent body of Y 980115 and would provide further clues of the reasons for its distinct features to CI-class meteorites.

**Fig. 3:**
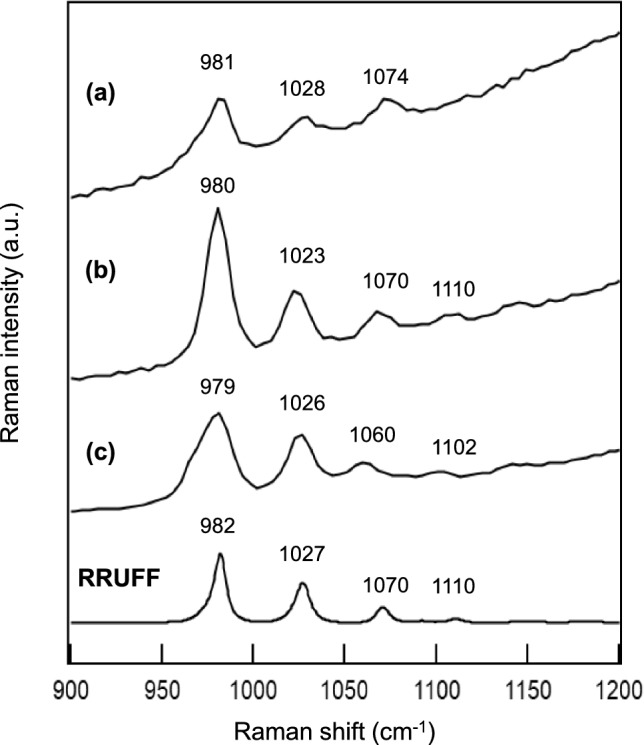
Comparisons of the observed Raman spectra of the grains of dehydrated magnesium phosphates Mg_3_(PO_4_)_2_, farringtonite, found on Y 980115 at PO_4_.^3−^-stretching vibrational modes and that for the reference of farringtonite (RRUFF, data ID: R130127)

Finally, since the study of Y 980115 on mineralogy and noble gas signatures suggested that the parent body of Y 980115 experienced incomplete dehydration [13], the degree of dehydration on phosphate minerals observed here is further discussed below. Four types of magnesium phosphates are generally known as completely dehydrated Mg_3_(PO_4_)_2_ (farringtonite), Mg_3_(PO_4_)_2_ ·4H_2_O (MO4), Mg_3_(PO_4_)_2_ ·8H_2_O (MO8, bobierrite), and Mg_3_(PO_4_)_2_·22H_2_O (MO22, cattiite). Their Raman and FT-IR spectra and thermodynamic natures were well studied [22, 23]. Owing to the sensitivities of Raman spectra to their structural difference, we can distinguish these four types of hydrated and/or dehydrated structures of magnesium phosphate minerals. The Raman shifts of the most intense peak appeared at around 960 cm^−1^ for the hydrated and dehydrated magnesium crystals are as follows: MO22; 936 cm^−1^, MO8; 960 cm^−1^, MO4; 963 cm^−1^, and dehydrated, 980 cm^−1^ [23]. Figure 3 shows the comparisons of the Raman spectra of three magnesium phosphate grains found on Y 980115 and the reference dehydrated magnesium phosphate (Farringtonite-I, RRUFF data, ID: R130127). We can clearly distinguish that the three magnesium phosphate grains found on Y 980115 in the present study are well dehydrated and completely different with MO4, MO8, and MO22 from the viewpoint of Raman shift of the most intensive peak. As mentioned, these features are observed when the magnesium phosphates including polymorphs of Farringtonite-II and –III experienced the heating up to above 750 degree Celsius (hydrated water molecules should be detached) and then cooled to the room temperatures [20]. Thus, we can support that the parent body of Y 980115 surely experienced thermal alteration at such high temperature [8–15, and references therein] also from the Raman spectroscopic study on magnesium phosphate grains. Further, it should be also noted that the bandwidths of the Raman peaks observed in the 980–1110 cm^−1^ for the magnesium phosphates found on Y 980115 show somewhat broadening compared to that of the reference farringtonite crystal (RRUFF data ID: R130127). These features might indicate the inhomogeneity of polycrystalline structure of the magnesium phosphate grains found on Y 980115, induced by the shock heating due to the collision of other planetesimals and the following cooling resulting in the polycrystalline phase on the parent body of Y 980115. Such collision events between planetesimals often occurred at the early stage of the solar system formation [24, 25].

## Conclusion

Minor grains of phosphate minerals found on Y 980115 meteorite were investigated by Raman microspectroscopy. Three grains, which are all of the found phosphate grains in the present study, showed characteristic series of Raman peaks above 980 cm^−1^ of the dehydrated form of magnesium phosphate Mg_3_(PO_4_)_2_ (“farringtonite I”). However, their bandwidths were somewhat broadened, indicating that they were formed via a tentative heating and following cooling processes. Crystal polymorphologies of farringtonite-II and -III are known to be irreversibly transform to Farringtonite-I by the heating of 750–800 degree Celsius. Thus, the Raman spectroscopic assignment of the phosphate grains strongly supports that the parent body of Y 980115 experienced thermal alteration with the temperature of up to 750–800 degree Celsius, which have been suggested by other TGA and XRD experiments. Since the crystal structures and the number of hydrated crystal water molecules in phosphate minerals sensitively reflect the aqueous and thermodynamic conditions where they form and experienced, Raman microspectroscopic study on phosphate minerals would provide another strong tool to analyze geochemical materials for elucidating their evolution and transformation occurred in terrestrial and space environments.

## Data Availability

The datasets analyzed during the current study are available from the corresponding author on reasonable request.
